# Mycophenolate-induced Colitis: A Case Report with Focused Review of Literature

**DOI:** 10.7759/cureus.6774

**Published:** 2020-01-25

**Authors:** Rehan Farooqi, Afrin Kamal, Carol Burke

**Affiliations:** 1 Internal Medicine, Medstar Union Memorial Hospital, Baltimore, USA; 2 Gastroenterology, Cleveland Clinic, Cleveland, USA

**Keywords:** colitis, mycophenolate induced colitis, crypt cell apoptosis, drug induced colitis, ulcerative colitis, inflammatory bowel disease (ibd)

## Abstract

Mycophenolate mofetil (MMF) is an immunosuppressive medication used for the management of various autoimmune diseases, and patients with bone marrow and solid organ transplants. Gastrointestinal side effects are seen 45% of the time and they include nausea (29%), vomiting (23%), constipation (38%), diarrhea (50%-92%), and colitis (9%). In 98% of cases, resolution of diarrhea occurs within 20 days upon discontinuation of the MMF. Data is scarce regarding approach in the treatment of MMF-induced colitis. We report a case of MMF-induced colitis diagnosed by colonoscopy and histopathology. This case illustrates the challenges encountered while managing MMF-induced colitis.

## Introduction

Mycophenolate mofetil (MMF) is an immunosuppressive medication commonly used to prevent rejection in solid organ transplant recipients. Active metabolite of MMF, mycophenolic acid, inhibits inosine monophosphate dehydrogenase which is the rate-limiting enzyme in purine synthesis for T and B-cell proliferation [1]. Enterocytes are 50% dependent on the de novo pathway of purine synthesis which is why they are vulnerable to MMF’s antimetabolic effects; this impedes the growth and replication of small bowel epithelial cells which leads to disruption of fluid absorption and diarrhea [2]. Injurious effects of MMF can be detected in the colon and include mucosal changes ranging from edema, erythema, erosions, and ulcerations. Histopathologic findings of MMF injury include crypt architectural distortion and crypt cell apoptosis [2]. The latency period between initiation of MMF exposure and onset of enterocolitis is between six months to 15 years with the average being around three years [3].

## Case presentation

A 68-year-old male with a history of recurrent deep vein thrombosis on warfarin, single lung transplant secondary to idiopathic pulmonary fibrosis on MMF (1000 mg twice a day) for eight months prior to admission, diverticulitis and recurrent diverticular bleed complicated by sigmoidectomy and right colectomy with ileo-colonic anastomosis, respectively, presented with 6-8 episodes of maroon-colored stools daily for the past two months. Over this time, the patient had several admissions at different hospitals requiring blood transfusions and endoscopic evaluations. At an outside hospital, esophagogastroduodenoscopy (EGD) demonstrated esophageal, gastric, and duodenal ulcers. He was treated with oral mesalamine for presumed inflammatory bowel disease. The patient was referred to the hospital for a second opinion after his symptoms did not improve.

**Figure 1:**
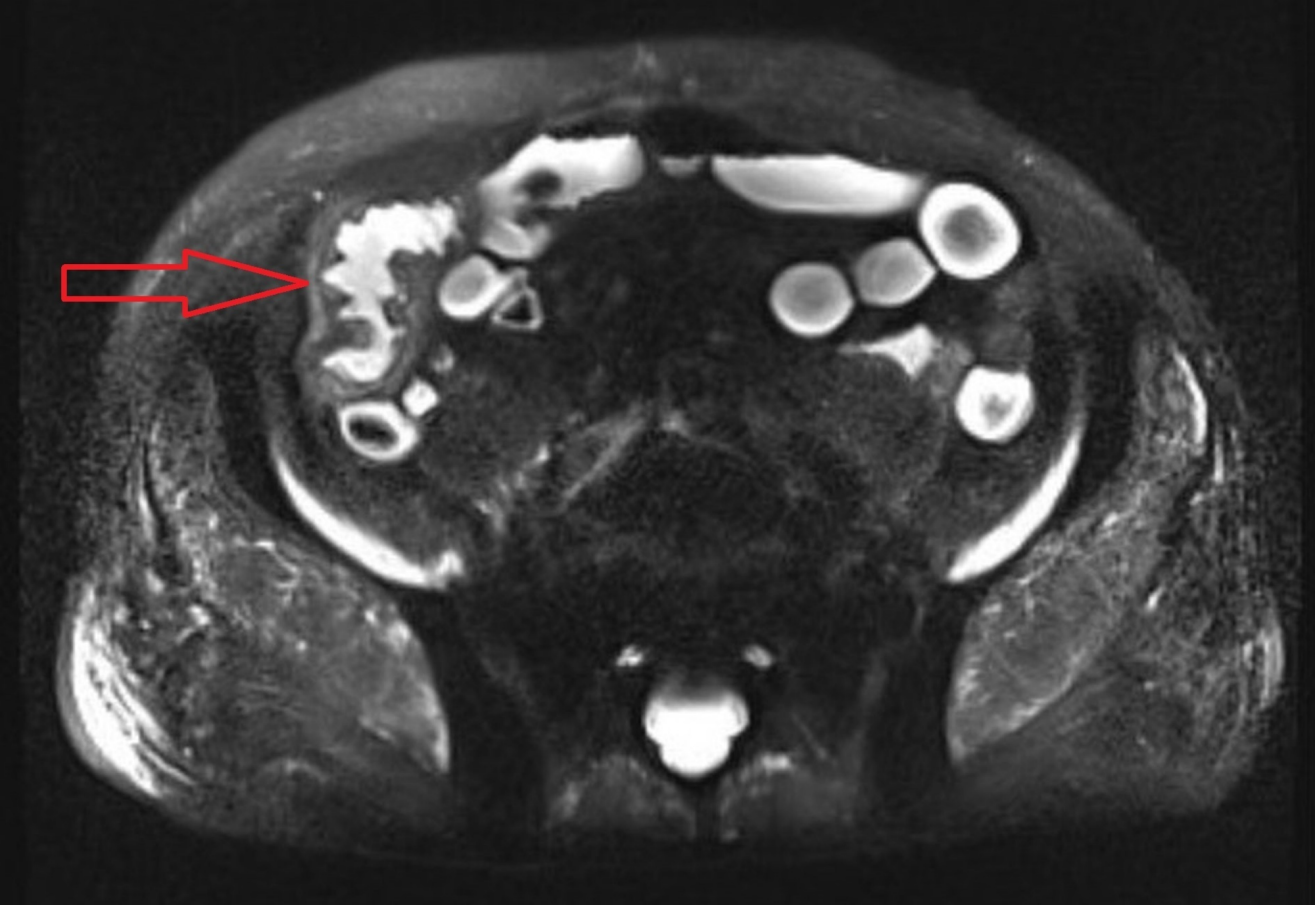
Magnetic resonance enterography depicting small bowel wall thickening, mural hyper enhancement and peri-enteric stranding involving 10-cm segment of the distal terminal ileum (red arrow)

Physical examination was significant for a chronically ill-appearing man with mild tenderness in the right lower quadrant of the abdomen and maroon colored stools on digital rectal exam. The rest of the examination was unremarkable. Laboratory studies revealed a hemoglobin level of 9.4 g/dL (normal range, 13.5-17.5 g/dL), iron 36 ug/dL (normal range, 41-186 ug/dL), ferritin 134.5 ng/mL (normal range, 30 - 565 ng/mL), and creatinine of 1.64 mg/dL (normal range, 0.73 - 1.22 mg/dL). Electrolytes, liver function, blood cytomegalovirus DNA, stool for ova & parasites, interferon-gamma release assay, and stool Clostridium difficile antigen were negative. A magnetic resonance enterography (MRE) of the abdomen and pelvis demonstrated small bowel wall thickening, mural hyper-enhancement, and peri-enteric stranding involving a 10-cm segment of the distal terminal ileum extending to the ileo-colonic anastomosis (Figure 1).

**Figure 2:**
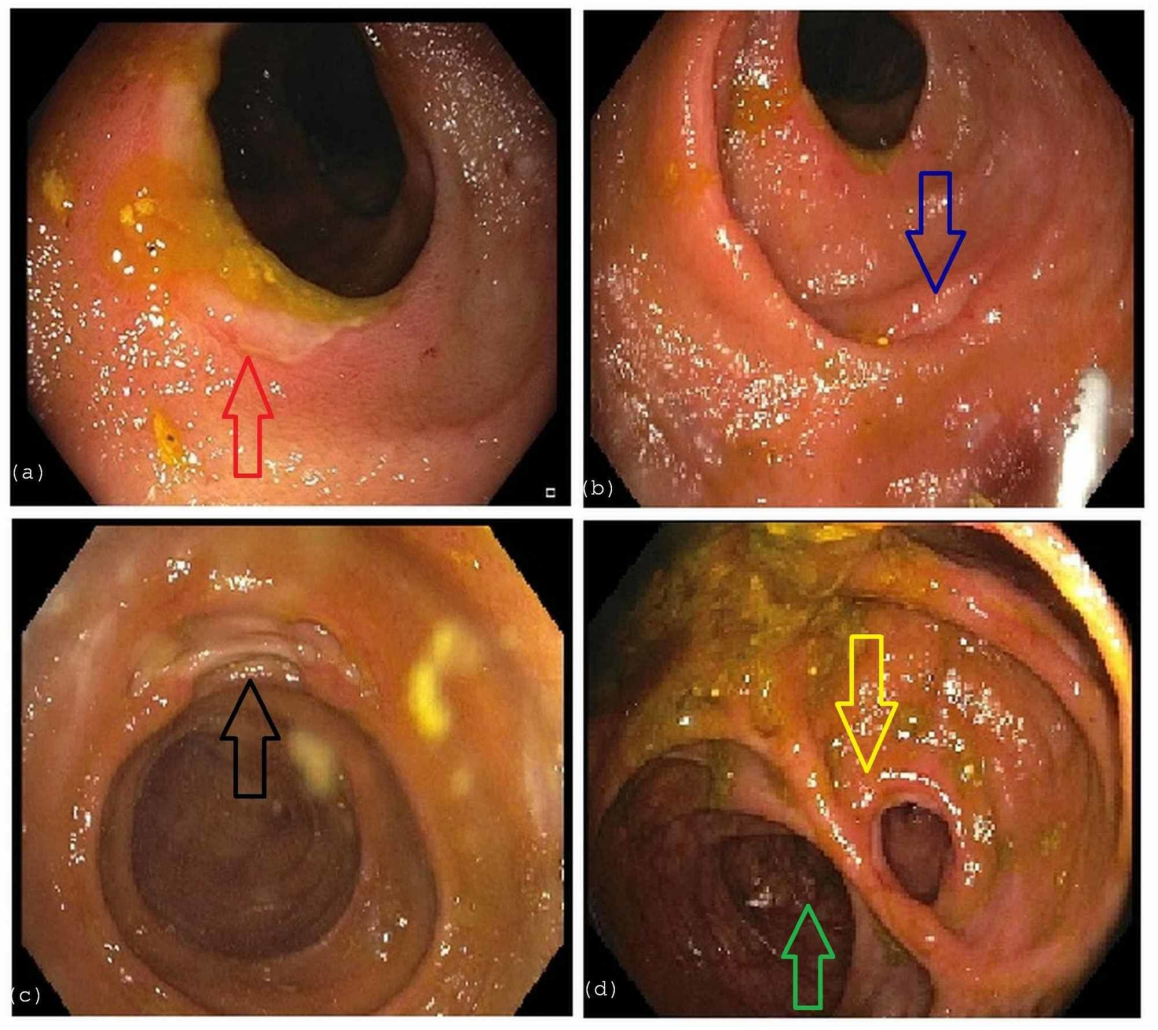
Colonoscopy a) depicts ulceration (red arrow) in the ascending colon. b,c) depict ulceration in the distal 15 cm of the neo-terminal ileum (blue and black arrows) with normal-appearing intervening mucosa. d) visualizes anastomosis of the distal ileum (yellow arrow) to the transverse colon (green arrow).

No active inflammation in the colon or rectum was noted. An EGD demonstrated a normal esophagus, stomach, and duodenum. Colonoscopy revealed numerous ulcers in the ascending colon, ileo-colonic anastomosis, and in the distal 15-cm of the neo-terminal ileum with normal-appearing intervening mucosa (Figure 2).

**Figure 3:**
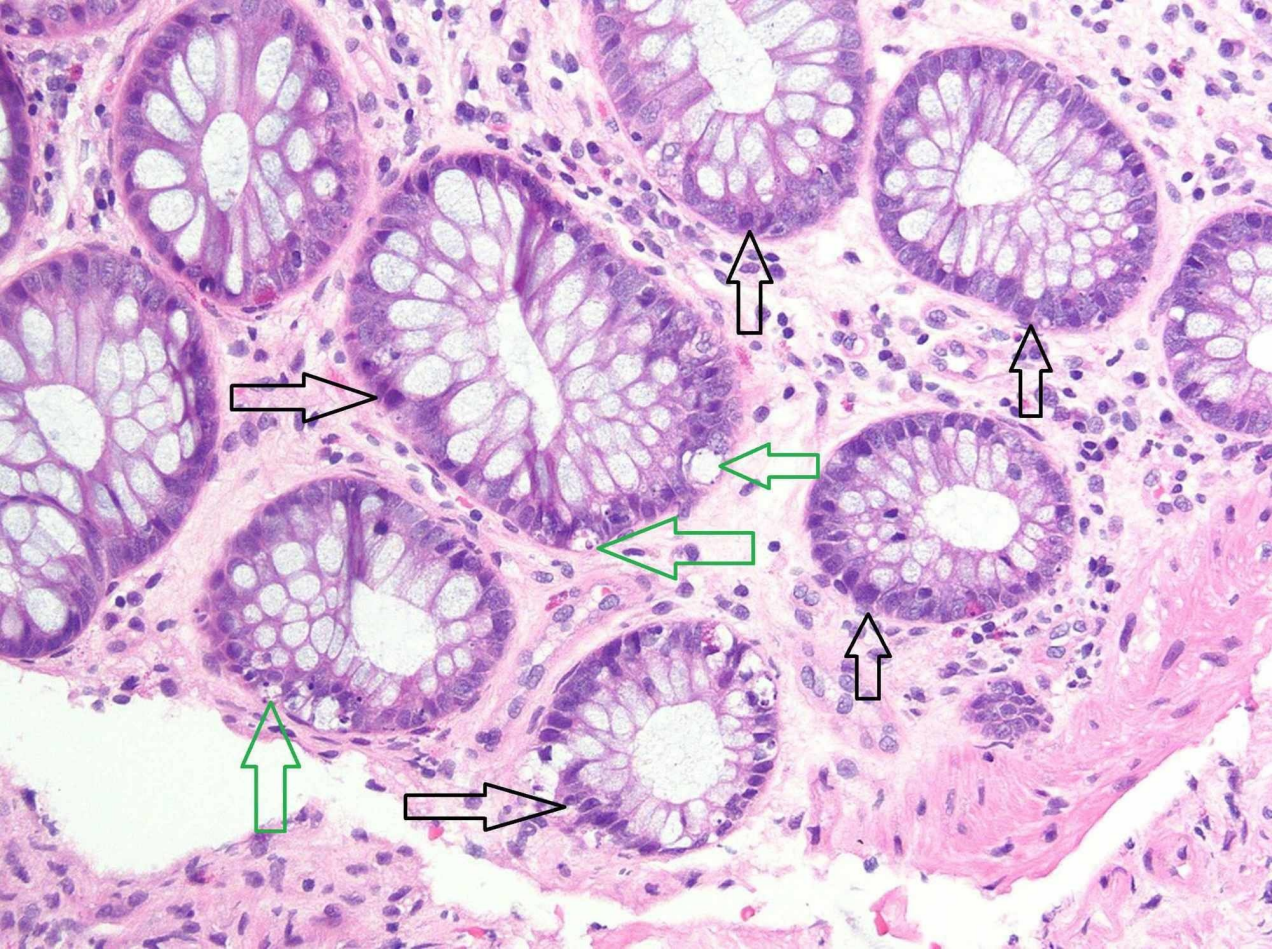
Photomicrograph from the colon biopsy showing architectural distortion with unevenly spaced lumen and crypts Several damaged crypts (black arrows) are present, scattered throughout the colonic mucosa (hematoxylin and eosin (H&E), original magnification x100). Also present are apoptotic bodies (green arrows) suggestive of cellular injury and turnover (H&E, original magnification x100). There is no evidence of active inflammation or viral cytopathic effect.

Biopsy of the ulcers with immunohistochemical staining for cytomegalovirus was negative. Histology revealed mild crypt architectural distortion with crypt cell apoptosis from the ascending colonic ulcers and patchy active ileitis from the ileo-colonic anastamotic ulcerations (Figure 3).

In view of MMF exposure and histopathologic findings of crypt architectural distortion and crypt cell apoptosis, our patient was diagnosed with MMF-induced enterocolitis and MMF was promptly discontinued.

**Figure 4:**
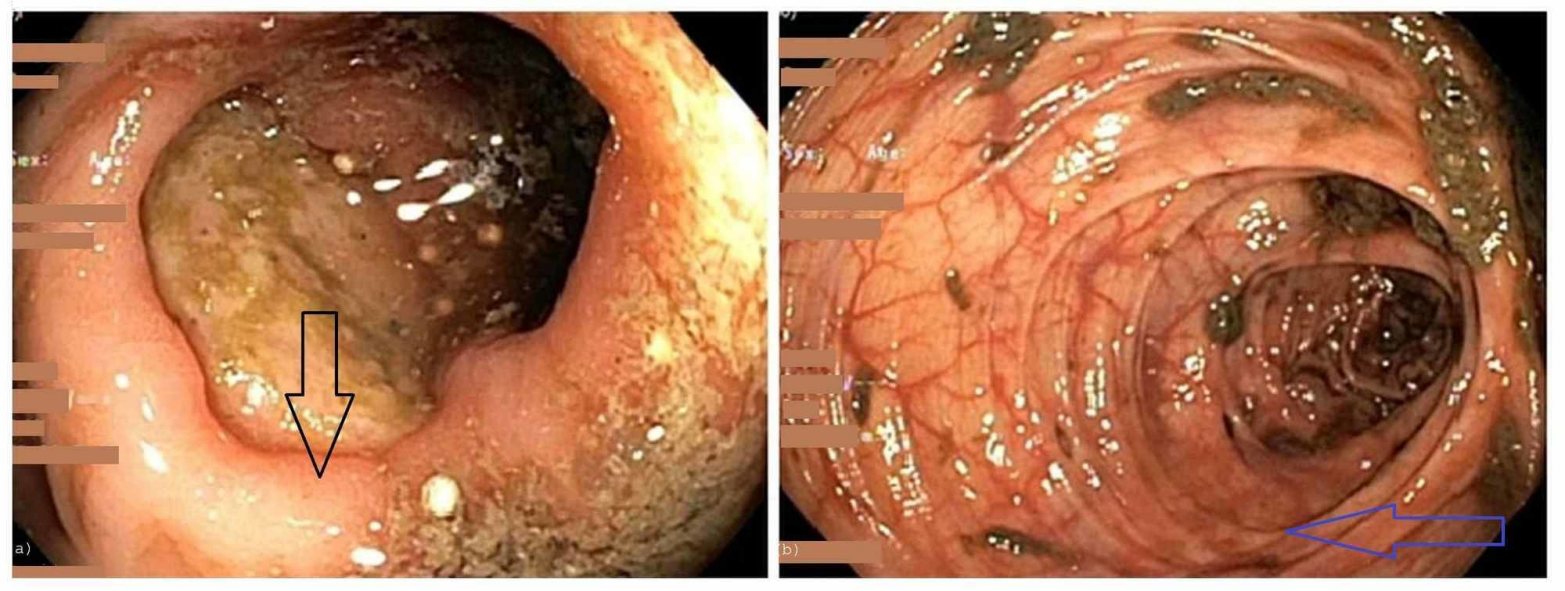
Repeat colonoscopy after five days of intravenous steroids showing significant mucosal improvement of the ascending colon (4a - black arrow), and transverse colon (4b - blue arrow)

Seventy-two hours after discontinuation of MMF, the patient continued to experience bloody diarrhea requiring RBC transfusions. Intravenous (IV) Solumedrol 20 mg every eight hours was initiated with mild improvement in the frequency of diarrhea. After five days of IV Solumedrol therapy, a repeat colonoscopy demonstrated evidence of complete healing of the numerous small ulcerations and improvement in the appearance of the large ulcerations, suggestive of overall endoscopic improvement (Figure 4).

The patient was transitioned to oral steroids and steadily experienced clinical improvement. Unfortunately, the patient’s hospital stay was complicated by hypoxic respiratory failure and he succumbed to death from aspiration pneumonia.

## Discussion

MMF is a commonly prescribed adjunct immunosuppressive agent in transplant therapy. Up to 45% of patients report diarrhea, vomiting, and abdominal pain; diarrhea being the most commonly reported side effect [4,5]. As an immunosuppressant, MMF can affect the entire gastrointestinal system leading to clinical complications ranging from esophagitis, gastroesophageal reflux disease, to enteritis and colitis. Development of diarrhea due to enteritis and/or colitis can be difficult to recognize as only 2%-9% of patients on MMF develop these complications [5,6]. Pathology can resemble inflammatory bowel disease, graft-versus-host disease (GVHD), acute colitis or ischemia [6]. There are treatment differences for each condition which is why obtaining biopsies is recommended to differentiate between these etiologies [7]. Specific histologic features of MMF related colitis include: crypt architectural disarray, increased lamina propria inflammation, dilated damaged crypts, increased crypt epithelial apoptosis and GVHD-like changes [8].

No guidelines are available to guide clinicians to treat MMF-induced enterocolitis. Several case reports have demonstrated that diarrhea improves within three to five days of discontinuing MMF [9-10]. One systemic review revealed that in 98% of the cases, diarrhea resolves within 20 days upon discontinuation of the MMF [3]. If symptoms are persistent despite MMF discontinuation, prednisone and/or infliximab has shown improvement. 

Table 1 lists the cases of MMF induced colitis and how these patients were managed [10-19]. In 12 of the 13 case reports found, patient's symptoms improved after lowering the offending agent dose or discontinuing the medication. Out of the 13, only two patients were already on steroids [13,14]. Out of the 13 cases, six of them underwent repeat colonoscopy at different times from the intervention to assess for healing [14-19]. Bouhbouh et al. gave a single infusion of 5 mg/kg of infliximab after previous futile attempts with MMF discontinuation, and 50 mg of prednisolone IV daily for two weeks [11]. Within three days, after a single infusion of infliximab, the stool frequency dropped significantly [11].

**Table 1:** Illustrates reported cases of mycophenolate-induced colitis to date with different management strategies that have been used; the table also indicates the timing of symptom improvement from the intervention Important to note that all patients underwent colonoscopy and/or flexible sigmoidoscopy for tissue pathology.

	Mycophenolate mofetil (MMF) dosing	Main symptom	Endoscopic findings	Histologic findings	Steroids given? (dosing)	Infliximab Given? (dosing)	Timing of symptom improvement
Bouhbouh (2010)	500mg BID	Watery, non-bloody diarrhea, abdominal pain, weight loss	Linear ulcerations throughout colon	Extensive ulceration with transmural mixed-cellular infiltration without granulomata	Yes. 2 weeks of Prednisone 30 mg PO daily, followed by 2 weeks of 25 mg prednisolone IV BID	Yes (5mg/kg)	72 hours after Infliximab
Johal (2014)	1,500 mg BID	Watery, non-bloody diarrhea, abdominal pain, weight loss	Segmental erythematous mucosa with ulcers in sigmoid, descending, splenic flexure and proximal transverse colon	Dilated crypts, eosinophilic epithelial changes, crypt abscesses with apoptotic bodies	No	No	5 weeks following MMF cessation
Goyal (2016)	Not provided	Watery, non-bloody diarrhea, abdominal tenderness and distention	Normal mucosa	Crypt atrophy, increased crypt apoptosis	No	No	3 days following MMF cessation
Jakes (2012)	750 mg bid	Abdominal pain and weight loss	Patchy inflammation of ascending colon, ileocecal valve was grossly thickened, stenosed, and ulcerated, consistent with a Crohn’s-like disease process.	Extensive ulceration	No	No	8 weeks following MMF reduction first to 250 mg bid and eventually discontinuing. Pt also underwent ex-lap s/p right hemicolectomy with no evidence of inflammatory changes within small or large bowel
Jakes (2012)	750 mg bid	Watery, non bloody diarrhea with large mucus	Severe pancolitis	Noncaseating granulomas within the lamina propria consistent with Crohns Disease	No	No	Resolution of colitis after MMF cessation, duration unknown
Jakes (2012)	180 mg bid	Profuse watery, non bloody diarrhea with right lower quadrant abdominal tenderness	Pancolitis with rectal sparing	Focal active colitis, no granulomas.	No	No	8 months after discontinuation of Myfortic, patient had sigmoidoscopy which showed no active inflammation. Unknown when patient noted improvement in symptoms
Moroncini (2018)	Not provided but started 2 months ago	Left sided abdominal pain, nausea, vomiting, and fever	Mucosal hyperemia, multiple serpiginous ulcers involving the transverse and descending colonic mucosa, with rectal sparing	ulceration, granulation tissue and hyalinised appearance of the mucosa and submucosa	No	No	5 days following MMF discontinuation. Repeat colonoscopy 1 month later showed complete resolution of ulcer
Tayyem (2018)	500 mg bid and Prednisone 15 mg daily	non-bloody diarrhea, dysphagia to solid food, nausea and unintentional weight loss of 2 weeks’ duration.	EGD: normal oesophagus, multiple small antral ulcers and reactive gastropathy. Colonoscopy: mucosal edema and erythema with small mucosal hemorrhages and punctate ulcerations in the ascending colon, patchy colitis in the transverse colon and rectal sparing	Colonic biopsies showed focal crypt abscesses (withered crypts) with occasional apoptosis of epithelial cells, frequent tingible body macrophages and eosinophils within the lamina propria	Patient was already on Prednisone 15 mg daily	No	5 weeks after MMF discontinuation
Gorospe (2012)	1000 mg bid	2-week history of profuse, watery diarrhoea that persisted through the night and with fasting	Flexible sigmoidoscopy showed mild erythema	apoptosis, crypt distortion and abscess; consistent with MMF-induced colitis	No	No	Five days later, the patient’s stool frequency decreased to twice daily until complete resolution. At 1 month follow-up, her MMF was restarted at a lower dose (500 mg/day) which was tolerated well without any recurrence of gastrointestinal issues.
Hamouda (2012)	Prednisone and MMF. Dosages not known	Profuse watery diarrhea, 6 to 8 times per day and weight loss	ulcerative diffuse colitis from the cecum to the rectum	mild crypt architectural distortion (Figure 1). The lamina propria showed edema and an increased number of inflammatory cells containing many neutrophils. Damaged crypts with mucus depletion and cryptitis. No granuloma	No	No	Symptoms regressed within 5 days after switching from MMF to azathioprine. Control colonoscopy showed reparative changes after 2 months
Kim (2000)	Dose not known but between 2 to 3 gm daily.	abdominal pain and watery diarrhea which progressed to bloody diarrhea	multiple ulcers and mucosal hyperemia and edema in the entire colon	Histology did not reveal viral cytopathic changes and immunohistochemical stains for cytomegalovirus infection were negative.	Patient was already on steroids	No	Abdominal pain and hematochezia improved rapidly. Follow-up colonoscopy 1 month later showed complete healing of previous lesions
Johal (2014)	1000 mg bid and increased to 1500 mg bid four months prior to presentation	Abdominal pain, nausea, intermittent bloating and profuse watery non bloody diarrhea.	segmental erythematous mucosa and multiple ulcers in the sigmoid colon, descending colon, splenic flexure and proximal transverse colon	dilated damaged crypts, eosinophilic epithelial changes and crypt abscesses with apoptotic bodies, a pattern of injury highly suggestive of MMF-related colitis	No	No	5 weeks after MMF discontinuation
Sonoda (2017)	1gm daily	Watery diarrhea which progressed to bloody diarrhea	multiple deep ulcers in the ileum	mild crypt distortion	No	No	Symptoms improved soon after MMF was discontinued. Six months later, the ileal mucosa was healed
­(Patra) 2012	Not provided	Significant weight loss, sitophobia for five months, and a recent onset of bleeding per rectum	Colonoscopy demonstrated ileal and cecal ulcers	Histopathology revealed crypt dropout, with focal disarray of the crypt architecture, along with apoptosis of the crypt epithelial cells. The crypt epithelial apoptotic rate was greater than 5 / 100 crypts. The lamina propria was edematous and showed focal collection of mild lymphomononuclear inflammatory cell infiltrate	Patient already on steroids, unknown dose	No	1 week after MMF cessation. Repeat colonoscopy after 1 month showed healing ulcers

The mucosal injury from MMF is thought to be related to the formation of immunotoxicologic reactions in the bowel, increased mucosal inflammation, and decreased mucosal protection [20]. The decreased mucosal protection is hypothesized secondary to the upregulation of intracellular phosphatidylcholines, prominent membrane phospholipid that maintains gastrointestinal barrier function, leading to disruption in membrane phospholipids and subsequently decreased mucosal defense [20]. One of the postulated mechanisms for mucosal inflammation is that the byproduct, acyl gluconoride, causes local irritation of the epithelium which then stimulates mononuclear cells to release tumor necrosis factor (TNF)- α, subsequently impacting the development of mucosal inflammation [20].

## Conclusions

Since the advent of post-transplant immunosuppression therapy, MMF-induced enterocolitis is uncommon with debilitating complications; limited data is available in the literature regarding the approach to treatment. There continues to be unanswered questions as to why some patients’ have refractory colitis, the benefits of oral or IV steroids, or biologic therapy (i.e. Infliximab), and the need for endoscopic reassessment for mucosal healing. With more cases being reported, we can better understand the natural course of the disease and help identify some of the answers. It is also prudent for physicians to inform the pathologist when a patient is on mycophenolate so the pathologist can be mindful of drug-induced colitis in the differential in addition to inflammatory bowel disease and GVHD.
